# Resveratrol inhibits the invasion and metastasis of colon cancer through reversal of epithelial-mesenchymal transition via the AKT/GSK-3β/Snail signaling pathway

**DOI:** 10.3892/mmr.2022.12870

**Published:** 2022-10-10

**Authors:** Li Yuan, Mengmeng Zhou, Dawei Huang, Harpreet S. Wasan, Kai Zhang, Leitao Sun, Hong Huang, Shenglin Ma, Minhe Shen, Shanming Ruan

Mol Med Rep 20: 2783–2795, 2019; DOI: 10.3892/mmr.2019.10528

Subsequently to the publication of the above article, an interested reader drew to the authors’ attention that the Nc-control and si-AKT1-control data panels featured in Fig. 4A for the SW480 Transwell assay experiments on p. 2788 appeared to contain overlapping data, such that the data, which were intended to show the results from experiments performed under different experimental conditions, may have been derived from the same original source. Furthermore, in Fig. 5 on p. 2789, there appeared to be some overlapping data comparing the AKT1 western blotting data with the p-AKT1 data, and the same data also appeared in Fig. 4D for the p-AKT1 data presented there.

The authors have re-examined their original data, and have realized that these figures were assembled incorrectly; essentially, the si-AKT1-control data panel was selected incorrectly for Fig. 4A, and the authors were able to retrieve the original data for the western blots presented in Fig. 5. The corrected versions of Figs. 4 and 5 are shown on the next page. The authors confirm that these errors did not have any major impact on the conclusions reported in their paper, and are grateful to the Editor of *Molecular Medicine Reports* for allowing them this opportunity to publish a Corrigendum. Furthermore, the authors apologize to the readership for any inconvenience caused.

## Figures and Tables

**Figure 4.**
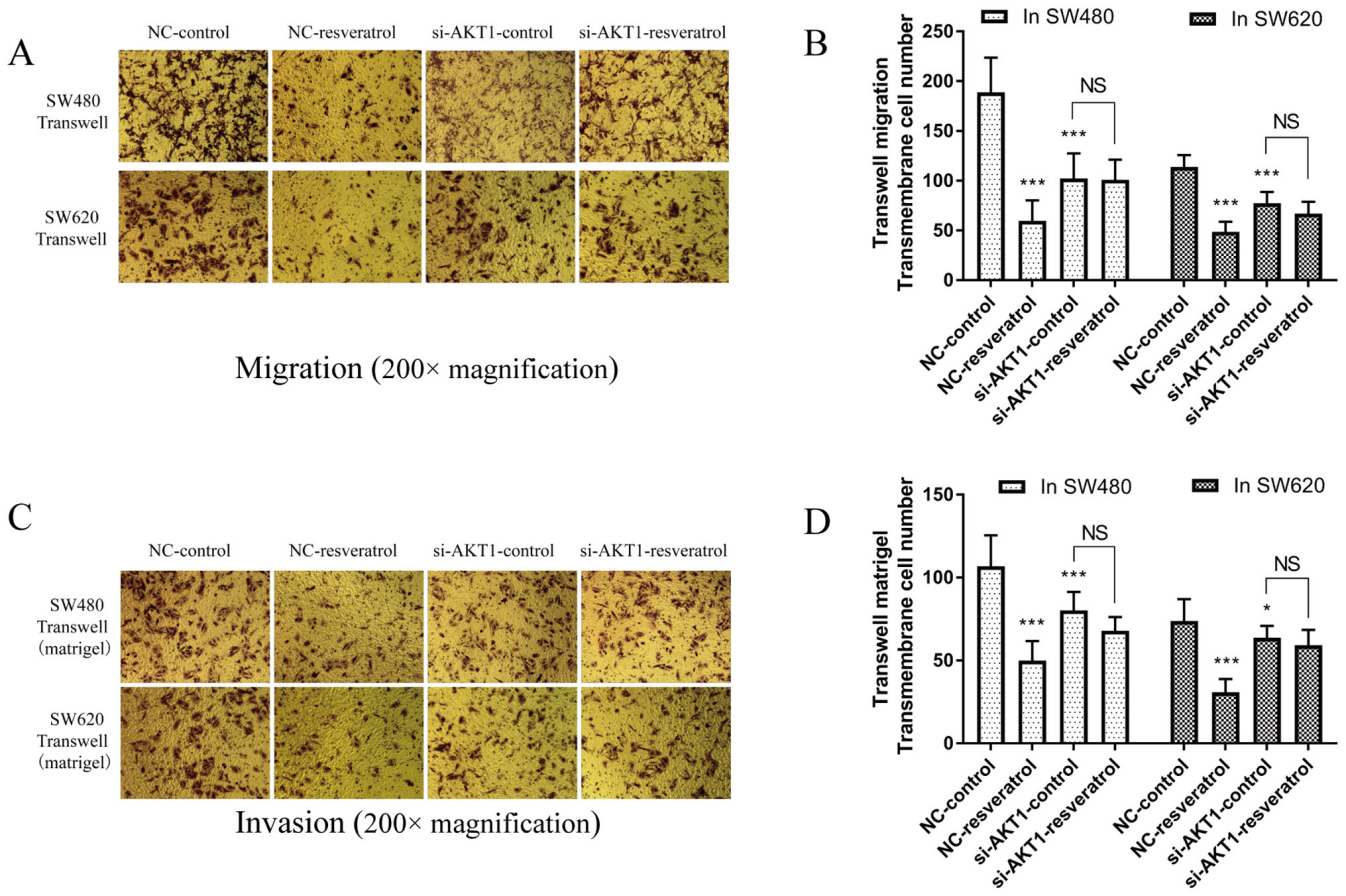
Resveratrol inhibits the migration and invasion of SW480 and SW620 cells *in vitro*. Cells were subjected to the same drug treatments as those used in the scratch wound healing assay. (A and B) Transwell migration assay revealing the migration of SW480 and SW620 cells subjected to various treatments. (C and D) Transwell invasion assay revealing the invasion of SW480 and SW620 cells subjected to various treatments. The number of migrated and invaded cells passing through the membrane in the NC-resveratrol and si-AKT1-control groups was significantly lower than that in the NC-control group. There was no significant difference between the number of migrated and invaded cells passing through the membrane in the si-AKT1-control and si-AKT1-resveratrol groups. ^*^P<0.05, ^***^P<0.001, and NS (P>0.05) compared with the respective control; n=9. These data were evaluated by one-way ANOVA. NC, negative control; si-, small interfering-; NS, not significant.

**Figure 5.**
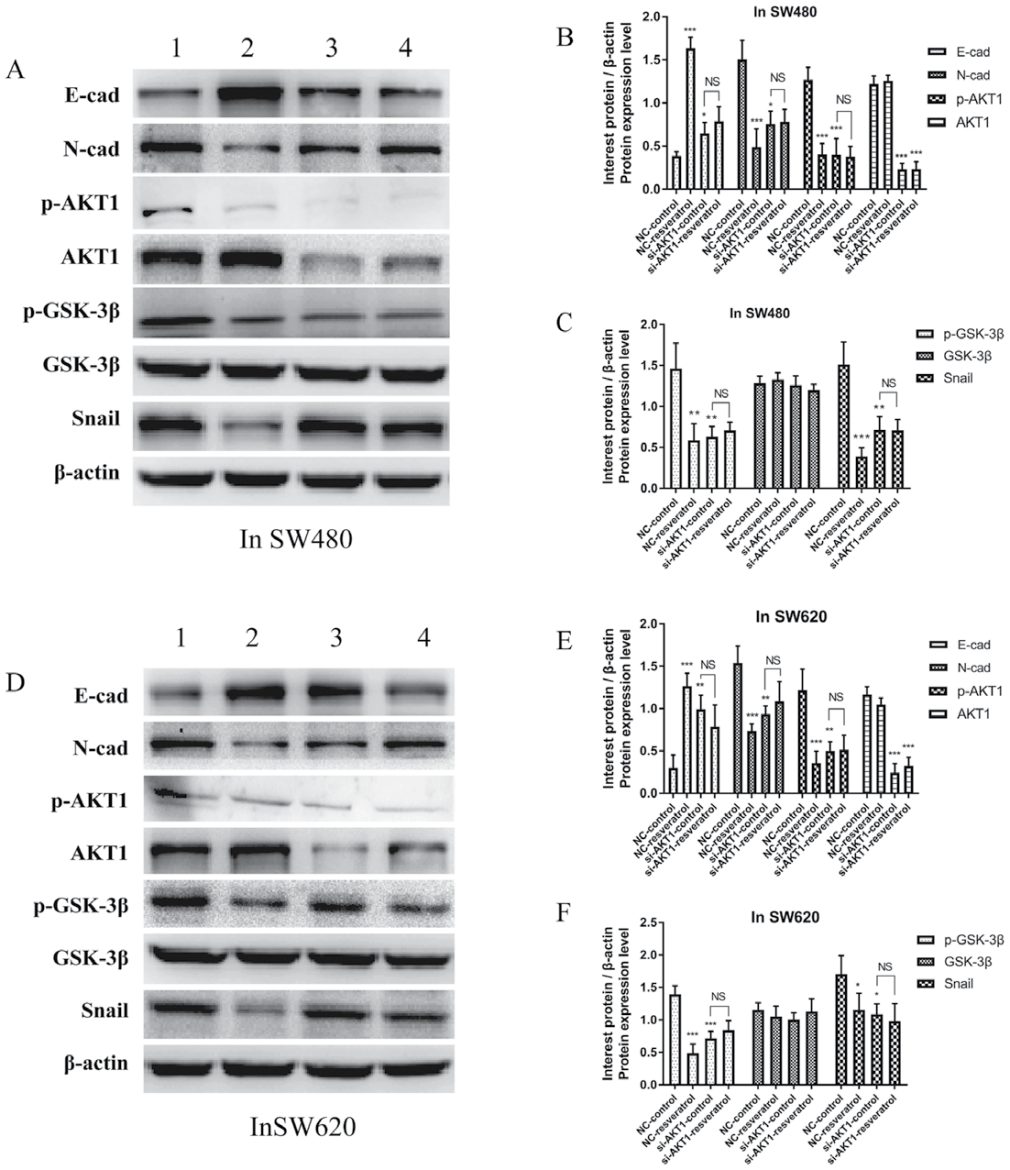
Resveratrol reverses EMT in colon cancer cells via regulation of the AKT/GSK-3β/Snail signaling pathway *in vitro* (1=NC-control, 2=NC-resveratrol, 3=si-AKT1-control, 4=si-AKT1-resveratrol group). Cells were subjected to the same drug treatments as those used in the scratch wound healing assay. Expression of E-cadherin, N-cadherin, p-AKT1, AKT1, p-GSK-3β, GSK-3β, and Snail was evaluated in (A-C) SW480 cells and (D-F) SW620 cells subjected to various treatments via western blotting. In comparison with that in the NC-control group, E-cadherin expression was upregulated in the NC-resveratrol and si-AKT1-control groups, whereas the expression of N-cadherin, p-AKT, p-GSK-3β, and Snail was downregulated. However, there was no difference in the expression of these proteins between the si-AKT1-resveratrol and si-AKT1-control groups. ^*^P<0.05, ^**^P<0.01, ^***^P<0.001, and NS (P>0.05) compared with the respective control; n=3. These data were evaluated by one-way ANOVA. EMT, epithelial-mesenchymal transition; GSK, glycogen synthase kinase; p-, phosphor-; NC, negative control; si-, small interfering-; NS, not significant.

